# Human papillomavirus-based vs. cytology for cervical cancer screening: a systematic review with meta-analysis

**DOI:** 10.61622/rbgo/2026rbgo130

**Published:** 2026-03-20

**Authors:** Kellen Crizel da Rocha, Everton Bruno Castanha, Maria Eduarda Minervino Elias, Celene Maria Longo da Silva

**Affiliations:** 1 Universidade Federal de Pelotas Pelotas RS Brazil Universidade Federal de Pelotas, Pelotas, RS, Brazil.

**Keywords:** Human papillomavirus DNA tests, Papanicolaou test, Uterine cervical dysplasia, Early detection of cancer, Public health

## Abstract

**Objective:**

To evaluate the performance of HPV-based testing compared to cytology in primary cervical cancer screening.

**Study selection:**

This meta-analysis included randomized trials comparing HPV-based screening with cytology in women aged 20–69 with at least one year of follow-up. Studies with overlapping populations, combining screening methods, lacking outcomes of interest, or study protocols were excluded. CENTRAL, PubMed/MEDLINE, and Embase were searched on April 12, 2025. The main outcome is the detection rate of cervical neoplasia grade 2 or worse (CIN2+).

**Data collection:**

Analyses followed Cochrane and PRISMA guidelines.

**Data synthesis:**

A total of 1,707 articles were identified. After removing duplicate records and excluding studies according to the pre-established criteria, 24 were selected for full-text review. Finally, eight were included in the statistical analysis, totaling 414,846 patients in a per-protocol assessment. The detection rate of CIN2+ by HPV-based tests was 61% higher compared to conventional or liquid-based cytology [RR 1.61; 95% CI 1.30, 1.98; p < 0.00001]. While our findings demonstrate the superior sensitivity of HPV testing, there are concerns about potential overdiagnosis and overtreatment. The absence of defined management protocols for HPV-positive patients contributes to this challenge. Therefore, risk-stratification strategies, such as HPV DNA genotyping, are essential to optimize clinical care and reduce these risks.

**Conclusion:**

HPV-based screening has a greater capacity to identify cervical neoplasia than cytology.

PROSPERO registry: ID CRD420251027259.

## Introduction

Cervical cancer remains a significant public health concern, particularly in low- and middle-income countries, where access to timely screening and treatment is often limited.^(1)^ Cytology-based screening, widely used globally, has proven effective in reducing mortality and enabling the early detection and treatment of precancerous lesions. However, this strategy has inherent limitations, such as variable sensitivity and reliance on subjective interpretation, which may lead to the underdiagnosis of high-grade squamous intraepithelial lesions (HSIL). The identification of these major cytological abnormalities requires follow-up with colposcopic evaluation, aiming to establish a definitive histological diagnosis of cervical intraepithelial neoplasia grade 2 or 3 (CIN2/3).^(2)^

The persistent infection with high-risk human papillomavirus (hrHPV) is recognized as fundamental for the development of cervical cancer.^(3)^ In this context, hrHPV testing, based on molecular detection of viral DNA, has emerged as a central tool for primary cervical cancer screening. Numerous clinical trials and population-based studies demonstrate that hrHPV testing is capable of identifying women at risk for CIN2+ lesions more directly and objectively than cytology, primarily due to its higher sensitivity. Several high-income countries have adopted HPV-based screening as a national strategy, and other regions are currently transitioning their programs. Although systematic evaluations and comprehensive comparisons of diagnostic performance have already been conducted, most included a mix of study designs.^(4)^ Our work focuses exclusively on randomized controlled trials, providing high-quality evidence that further strengthens and contextualizes the established knowledge.

This meta-analysis includes data from many studies representing a broad spectrum of geographic regions, healthcare systems, and screening approaches, offering robust evidence for comparing the effectiveness of HPV-based and cytology-based screening methods. Thus, this meta-analysis intends to evaluate the diagnostic accuracy of HPV-based screening in comparison with the routinely used cytology, considering key performance indicators and variations across different populations and screening contexts.

## Methods

This systematic review and meta-analysis was conducted following the methodological recommendations of the Cochrane Handbook for Systematic Reviews of Interventions and reported following the PRISMA (Preferred Reporting Items for Systematic Reviews and Meta-Analyses) guidelines.^(5)^ It was registered in PROSPERO with the ID CRD420251027259.

We included studies that met the following eligibility criteria: (1) randomized controlled trials; (2) comparing HPV-based screening with conventional or liquid-based cytology; (3) in women between 20 and 69 years of age; (4) with follow-up for at least one year; and (5) published studies. We excluded studies with (1) a combination of methods in primary screening; (2) overlapping patient populations; (3) no outcomes of interest; and (4) protocols. Only the study with the largest number of patients was included in overlapping populations.

Cochrane Central Register of Controlled Trials (CENTRAL), PubMed and Embase were systematically searched on April 12, 2025. The search strategy included the following terms: "human papillomavirus-based screening", "HPV-based screening", "HPV screening", "human papillomavirus DNA", "human papillomavirus-test", "HPV DNA test", "human papillomavirus testing-based", "human papillomavirus cervical screening", "human papillomavirus DNA tests", "cervical cancer", "uterine cervical neoplasms", "cervix neoplasms", "cervical neoplasms". The Cochrane Highly Sensitive Search Strategy for identifying randomized trials was used. We adapted this strategy for Embase using Query translator and removed the specific terms to search in CENTRAL (Supplementary Material - Chart 1S).

The study selection process was conducted independently by two reviewers, who screened all titles and abstracts using a standardized Excel template with predefined eligibility criteria. Full texts of potentially relevant studies were then assessed for inclusion. Discrepancies at either stage were resolved by a third reviewer, blinded to the initial decisions.

For data extraction, one reviewer collected information from the included studies using the same standardized Excel template, covering (1) country; (2) number of patients in intervention and control groups; (3) mean age of participants; (4) follow-up duration (months); (5) type of HPV-DNA test (self-collected or clinician-collected); and (6) detection rate of cervical intraepithelial neoplasia grade 2 or worse (CIN2+). A second reviewer independently verified all extracted data. Any disagreements were resolved through discussion with the third reviewer. No automated tools were used in this process.

Data were extracted from individual studies, focusing primarily on outcomes related to the detection of cervical intraepithelial neoplasia grade 2 or worse (CIN2+). For binary outcomes (e.g., CIN2+ detection), we calculated pooled relative risks (RRs) with 95% confidence intervals (CIs). When applicable, weighted mean differences (WMD) were used for continuous variables. Heterogeneity across studies was assessed using the Cochran Q test and I² statistic; p-values < 0.10 and I² > 25% were considered indicative of significant heterogeneity. All statistical analyses were performed using Review Manager (RevMan) version 5.4 (The Cochrane Collaboration, Copenhagen, Denmark) using a random-effects model. Risk assessment of individual studies was assessed using QUADAS-C,^(6)^ independently by two reviewers, and publication bias was assessed using funnel plots and Egger's test. GRADE assessment was applied to determine the evidence certainty.^(7)^

## Results

**Figure 1:**
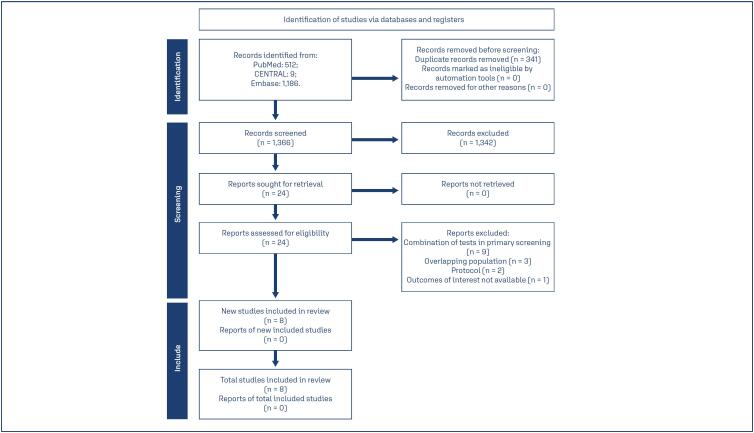
Study screening and selection

A total of 1,707 articles were identified. After removing duplicate records, 1,366 studies were screened for eligibility. Of these, 24 remained and were fully reviewed based on predefined eligibility criteria. Finally, eight met the inclusion criteria and were included in the statistical analysis (Figure 1). Among the included studies, three employed self-collected HPV DNA testing. For the cytology arms, three studies utilized liquid-based cytology, five used conventional cytology, and one used both (Chart 1). Overall, data from 414,846 patients were analyzed in a per-protocol assessment. The participants’ age and follow-up periods are specified in chart 1.(^(8)^

**Chart 1:** Baseline characteristics of individual studies

Study		Fujita et al. (2024)^(14)^	Gustavsson et al. (2018)^(15)^	Ogilvie et al. (2018)^(16)^	Isidean et al. (2016)^(17)^	Leinonen et al. (2012)^(18)^	Lazcano-Ponce et al. (2011)^(19)^	Nygård et al. (2022)^(20)^	Zhang et al. (2020)^(21)^
Country		Japan	Sweden	Canada	Canada	Finland	Mexico	Norway	China
Number of patients	I	1190	7997	9552	10120	66410	9202	77207	33561
	C	779	6364	9457	9991	65784	11504	80240	16035
	T	1969	14361	19009	20111	132195	20256	157447	60732
Eligible age group		30-58	30-49	25-65	30-69	25-67	25-66	34-69	35-64
Intervention		HPV-DNA for primary screening*	HPV-DNA for primary screening*	HPV-DNA for primary screening	HPV-DNA for primary screening	HPV-DNA for primary screening	HPV-DNA for primary screening*	HPV-DNA for primary screening	HPV-DNA for primary screening
Control		LB and conventional cytology	Cytology	LB-cytology	Cytology	Cytology	Cytology	LB-cytology	Cytology
Mean follow-up#		30	18	48	16.6 (M); 12.9 (SJ)	43.2	NA	18	24
Mean age	I	44.6	39.7	45	30-49 (73%)**	35-65 (83.1%)**	25-34 (44%)**	49.8	47
	C	44.5	39.5			35-65 (83.1%)**	25-34 (45%)**	49.9	

(I) Intervention Group; (C) Control Group; (T) Total number of patients; (LB) Liquid-based; (M) Montreal; (SJ) Saint John's;

* Self-sampling test;

** Age group, (%); # Months.

**Figure 2:**
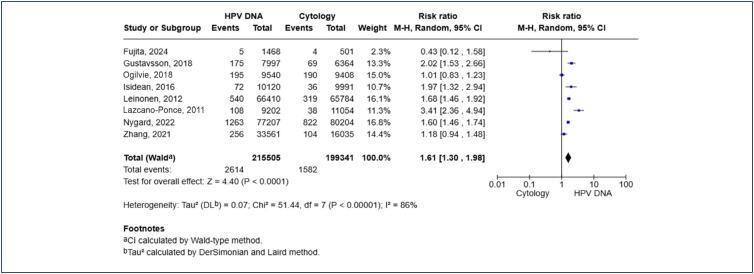
Pooled risk ratios of the detection rate of CIN2+

There were 215,505 patients in the HPV DNA group, with 2,614 pooled cases of CIN2+ lesions or worse, corresponding to 1.21% of this population. The cytology group had 199,341 patients, with 1,582 pooled cases of CIN2+, corresponding to 0.79% of this population. Then HPV DNA test demonstrated superior effectiveness in the detection of CIN2+ lesions compared to cytology [RR 1.61; 95% CI 1.30, 1.98; p < 0.00001] (Figure 2). However, there was a high level of heterogeneity in this analysis (I² = 86%; p < 0.0001), likely attributable to significant differences in the clinical follow-up protocols among the studies.

**Figure 3:**
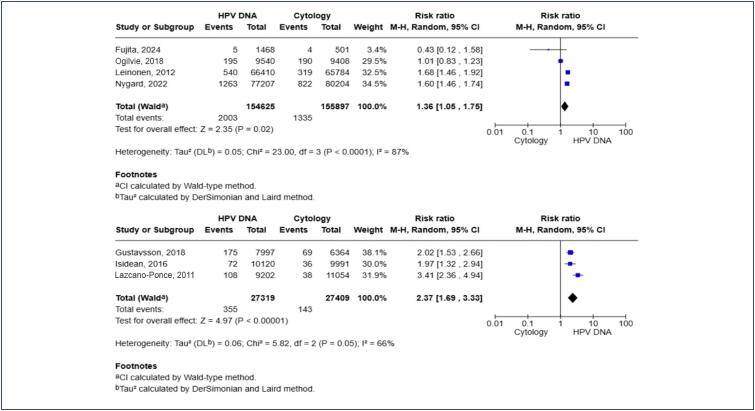
Subgroup analysis with studies that share similar protocols

**Figure 4:**
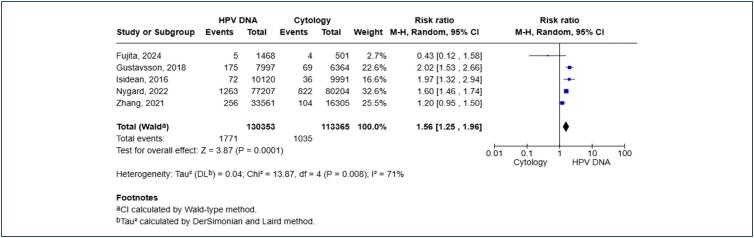
Subgroup analysis with studies that included only women aged 30 or more

Due to the observed heterogeneity, subgroup analyses were undertaken on studies sharing similar protocols (Figure 3). In the subgroup of studies that utilized cytology triage to refer patients with a positive HPV DNA test to colposcopy, HPV DNA remained a more sensitive test for CIN2+ lesions [RR 1.36; 95% CI 1.05, 1.75; p < 0.02]. However, this analysis continued to exhibit a high level of heterogeneity. Similar results were obtained in the subgroup where patients were referred directly to colposcopy [RR 2.37; 95% CI 1.69, 3.33; p < 0.00001], though this analysis also retained high heterogeneity. These results were also not altered when studies including women under the age of 30 were excluded, accounting for the transitional nature of HPV infection in younger women [RR 1.56; 95% CI 1.25, 1.96; p < 0.00001] (Figure 4). Additionally, a leave-one-out sensitivity analysis was conducted, and the results remained consistent and statistically significant after the sequential exclusion of each study.

### Study or Subgroup

**Figure 5:**
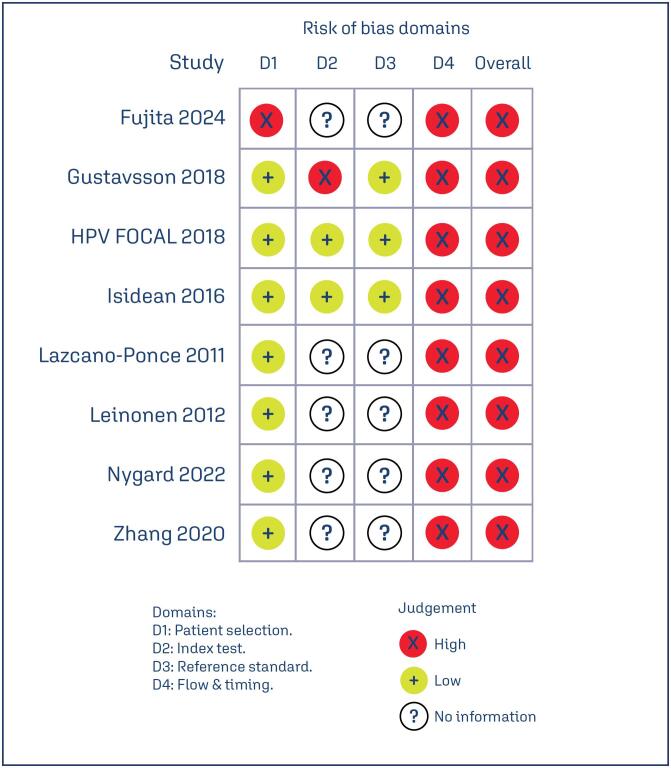
Risk of Bias (QUADAS-C)

**Figure 6:**
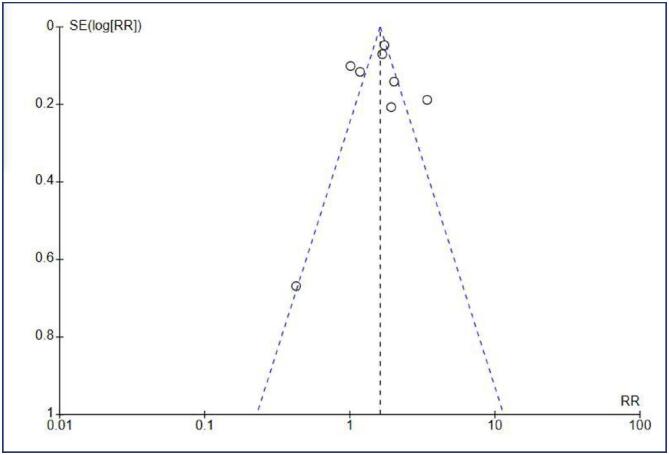
Funnel plot

**Figure 7:**
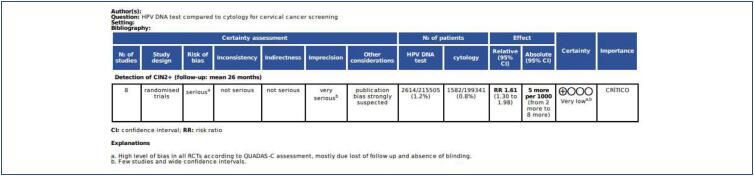
Grade assessment

The methodological quality and risk of bias for each included study were assessed using QUADAS-C (Figure 5). All studies in this meta-analysis have a high overall risk of bias. This could be explained by a large differential participation rate between groups and subsequent losses to follow-up, significantly impacting the comparability of detection rates between study arms. Furthermore, a majority of studies did not implement blinding for participants or for clinicians and pathologists, which also contributed to the overall risk of bias. Regarding publication bias, an assessment using a funnel plot (Figure 6) revealed a notable accumulation of studies on the right side of the graphic. While this asymmetry could indicate a potential for publication bias or a limited understanding within this research area, the small number of included studies (n < 10) did not allow a definitive conclusion. These factors, combined with a large confidence interval, led to a very low certainty in GRADE assessment (Figure 7).

## Discussion

In this systematic review and meta-analysis of eight randomized controlled trials involving 414,846 patients, the performance of the HPV DNA test was compared with conventional or liquid-based cytology in primary cervical cancer screening. The HPV DNA test demonstrated superior sensitivity in the detection of pre-neoplastic lesions; specifically, the pooled analysis reveals a significantly higher detection rate of CIN2 or worse with HPV-based screening, increasing diagnosed cases by 61%. While individual randomized controlled trials yielded mixed results regarding the best modality for primary cervical cancer screening, the pooled analysis presented in this meta-analysis provides more definitive evidence. This finding reinforces the substantial benefits of adopting the HPV DNA test in screening programs worldwide, as it significantly enhances the chances of an early diagnosis, which is fundamental for improving patient prognosis.^(14-21)^

From a clinical perspective, the higher detection rate of CIN2+ by HPV-based testing implies improved early identification of women at risk for cervical cancer, potentially leading to timely intervention and a reduction in the incidence of invasive cancer. Beyond this primary benefit, studies have also indicated that HPV DNA testing is associated with a lower incidence of vaginal and vulvar cancers. This secondary benefit can be plausibly explained by two primary mechanisms: (1) the superior sensitivity of the HPV DNA test for detecting CIN2+ lesions allows for more patients to receive treatment for cervical precursors, thereby potentially reducing the risk of persistent infection or progression and spread of HPV to other anogenital sites; and (2) the increased referral of HPV-positive individuals for colposcopy provides an additional opportunity to detect and subsequently treat vaginal and vulvar precancers that might otherwise be missed.^(22)^ Additionally, HPV DNA negative testing facilitates extended screening intervals, which can reduce patient discomfort associated with sample collection. However, some authors have raised concerns about the potential for loss of follow-up during these longer periods between gynecological visits.^(23)^

Although a key concern associated with highly sensitive screening tests, such as the HPV DNA test, is the potential for increased overdiagnosis and overtreatment. This is particularly relevant for younger women, typically under 30 years of age, where HPV infection is highly prevalent but often transient. In this demographic, high rates of HPV positivity can frequently lead to a large number of false positives and unnecessary follow-ups, given the considerably lower incidence of cervical cancer in this population.^(24,25)^ This meta-analysis included studies that referred patients directly to colposcopy after a positive HPV DNA test, and the ones who advocated for a triage strategy involving cytology. A third strategy, based on HPV genotyping, stratifies patients by risk, and it has been used in studies that evaluate HPV based screening in a real-life scenario. In this protocol, patients who tested positive for HPV types 16 and 18 were led immediately to colposcopy, while those with other high-risk HPV types may be managed with follow-up cytology and HPV DNA testing at 12 months. This flowchart is based on HPV 16 and 18 higher risk for CIN2+, the risk stratification for women under 30 years old, and the screening simplification.^(26)^ Biomarkers such as p16/Ki-67 immunohistochemistry have been suggested, but it's role in cervical cancer screening is not established.^(27)^

Our study has important limitations. Primarily, we observed heterogeneity in the main outcome and secondary analysis. This can be attributed to significant differences in the clinical follow-up protocols among the included studies. For example, Gustavsson et al.,^(15)^ Isidean et al.,^(17)^ and the Lazcano-Ponce et al.^(19)^ referred patients to colposcopy after one or two positive HPV DNA test results. In contrast, Zhang et al.^(21)^ randomized patients in a 1:1:1 proportion to direct colposcopy, cytology triage, or VIA/VILI exam, particularly relevant in a rural population setting. Other studies adopted a strategy where patients with a positive HPV DNA test underwent a cytology test before colposcopy. To address this heterogeneity, subgroup analyses were performed based on similar follow-up protocols, but substantial heterogeneity persisted in both subgroups. The study by Zhang et al.^(21)^ was excluded from these subgroup analyses due to the unavailability of specific data regarding patient distribution across its various management arms. Considering the transitional nature of HPV infection in younger women, a subgroup analysis excluding studies with women under 30 years of age was also conducted, but heterogeneity again persisted.

Beyond differences in clinical follow-up protocols and age groups particularities, other factors can explain this results: (1) variations in the demographic characteristics of the screened populations, such as age distribution and baseline prevalence of HPV infections; (2) methodological variances among studies, such as the type of HPV DNA test used (self-collected versus clinician-collected); (3) different cytology methods (liquid-based versus conventional) in the control arms; (4) lack of blinding for participants, clinicians, and pathologists in some studies, which may have introduced detection and performance biases, as highlighted by our QUADAS-C assessment. The main compromised domain was the fourth, which concerns patient flow and follow-up. Finally, differences in national screening guidelines and healthcare system infrastructures across the diverse geographical regions where the studies were conducted could also account for the unexplained heterogeneity.

The primary outcome of this meta-analysis is the detection rate of CIN2+ lesions, which are precursors to invasive cervical cancer. Although this is a relevant clinical diagnosis requiring early medical intervention to prevent unfavorable outcomes, these numbers do not directly correspond to the incidence of invasive cancer and cervical cancer mortality, which would be most relevant in terms of public health. While the reduction in the incidence of these two events with primary HPV DNA testing is documented,^(28)^ there is also the risk that lesions that would spontaneously regress may be detected, leading to unnecessary clinical follow-up and patient anxiety. The follow-up time was also insufficient to evaluate major outcomes, since the longest trial had 4 years of clinical observation.

Furthermore, in all clinical trials, there was a high rate of loss to follow-up in intervention and control groups, often differing significantly, which certainly influenced the final results. Finally, we have a small number of included studies (n = 8), which makes it difficult to draw definitive conclusions. The risk of bias in our publication, as analyzed through a funnel plot, also showed an accumulation of studies on the right side of the graph, suggesting that smaller studies with non-significant or negative results may have remained unpublished, which could have affected our final analysis.

## Conclusion

In conclusion, HPV-based primary screening demonstrates superior sensitivity in the detection of CIN2 or worse lesions when compared with cytology, leading to a higher number of early diagnoses of cervical cancer precursors. However, this heightened sensitivity may also contribute to concerns regarding overdiagnosis and overtreatment. HPV DNA genotyping helps clinicians reserve more aggressive interventions for the most oncogenic types, resulting in more precise management strategies and preventing unnecessary procedures.

## Data Availability

The research data are described in the article presented.
