# Supplementary material for: Human papillomavirus-based vs. cytology for cervical cancer screening: a systematic review with meta-analysis
Source: Rev Bras Ginecol Obstet. 2026 Mar 20;48:e-rbgo130. doi: 10.61622/rbgo/2026rbgo130 (PMC13078516; doi:10.61622/rbgo/2026rbgo130)
Supplement: Supplementary Material [file 1806-9339-rbgo-48-e-rbgo130-suppl01.pdf]

Supplementary Material

Chart 1S. Search strategy

Pubmed: («human papillomavirus-based screening»[All Fields] OR «HPV-based screening»[All Fields] OR «HPV screening»[All Fields] OR «Human papillomavirus DNA»[All Fields] OR «Human papillomavirus-test»[All Fields] OR «HPV DNA test»[All Fields] OR «Human papillomavirus testing-based»[All Fields] OR «Human papillomavirus cervical screening»[All Fields] OR «human papillomavirus dna tests"[MeSH Terms]) AND ("Cervical cancer"[All Fields] OR "cervix neoplasm"[All Fields] OR "uterine cervical neoplasms"[MeSH Terms]) AND ("randomized controlled trial"[Publication Type] OR "controlled clinical trial"[Publication Type] OR "Randomized"[Title/Abstract] OR "placebo"[Title/Abstract] OR "drug therapy"[MeSH Subheading] OR "randomly"[Title/Abstract] OR "trial"[Title/Abstract] OR "groups"[Title/Abstract])
Embase: ('human papillomavirus-based screening' OR 'hpv-based screening' OR 'hpv screening' OR 'human papillomavirus dna' OR 'human papillomavirus-test' OR 'hpv dna test' OR 'human papillomavirus testing-based' OR 'human papillomavirus cervical screening' OR 'Human papillomavirus ONA test') ANO ('cervical cancer' OR 'uterine cervical neoplasms' OR 'cervix neoplasms' OR 'cervical neoplasms' OR 'uterine cervix cancer') ANO ('randomized controlled trial':it OR 'controlled clinicai trial':it OR 'randomized':ti,ab,kw OR 'placebo':ti,ab,kw OR 'drug therapy' OR 'randomly':ti,ab,kw OR 'trial':ti,ab,kw OR 'groups':ti,ab,kw).
CENTRAL: ('human papillomavirus-based screening' OR 'hpv-based screening' OR 'hpv screening' OR 'human papillomavirus dna' OR 'human papillomavirus-test' OR 'hpv dna test' OR 'human papillomavirus testing-based' OR 'human papillomavirus cervical screening' OR 'Human papillomavirus ONA test') ANO ('cervical cancer' OR 'uterine cervical neoplasms' OR 'cervix neoplasms' OR 'cervical neoplasms' OR 'uterine cervix cancer') ANO ('randomized controlled triai' OR 'controlled clinicai triai' OR 'randomized' OR 'placebo' OR 'drug therapy' OR 'randomly' OR 'triai' OR 'groups').
